# Impact of Bronchiectasis on Healthcare Resource Utilization and Direct Medical Costs of Managing Comorbid Chronic Obstructive Pulmonary Disease, Asthma, and Rheumatoid Arthritis in the United States

**DOI:** 10.1111/crj.70150

**Published:** 2025-12-25

**Authors:** Maitreyee Mohanty, Fei Tang, John Fastenau, Joseph Feliciano, Melanie Lauterio, Sebastian Fucile, Mahroz Haider, Ruxana T. Sadikot

**Affiliations:** ^1^ Insmed Incorporated Bridgewater New Jersey USA; ^2^ Cytel Waltham Massachusetts USA; ^3^ University of Nebraska Medical Center Omaha Nebraska USA; ^4^ VA Nebraska Iowa Healthcare System Omaha Nebraska USA

**Keywords:** asthma, bronchiectasis, comorbidity, COPD, health care costs, retrospective study, rheumatoid arthritis

## Abstract

**Background:**

Although patients with bronchiectasis often have comorbidities, the impact of bronchiectasis on managing these is unknown. This study assessed the incremental burden of managing chronic obstructive pulmonary disease (COPD), asthma, and rheumatoid arthritis in patients with bronchiectasis.

**Methods:**

This retrospective cohort study using Merative MarketScan US claims data included patients with a COPD, asthma, or rheumatoid arthritis diagnosis between January 2017 and December 2021. Within these cohorts, patients with a bronchiectasis diagnosis (excluding cystic fibrosis) were compared with nonbronchiectasis controls following 1:1 propensity score matching (1:2 for rheumatoid arthritis). Comorbid disease‐specific inpatient, outpatient, and emergency room (ER) visits and direct medical costs were reported.

**Results:**

After matching, 4291 patients with COPD, 2460 with asthma, and 566 with rheumatoid arthritis, all with bronchiectasis, and the corresponding controls, were included. For patients with COPD, proportions with COPD‐related outpatient (66.5% vs. 56.8%), ER (7.5% vs. 5.8%), and inpatient visits (4.5% vs. 3.1%), as well as respiratory‐related ($11 054 vs. $6961) and disease‐specific ($1384 vs. $1107) costs were significantly higher in the bronchiectasis cohort (vs. control cohort). For patients with asthma, asthma‐related outpatient visits (52.0% vs. 41.1%), respiratory‐related ($10 327 vs. $5458), and disease‐specific ($489 vs. $221) costs were significantly higher in the bronchiectasis cohort. For patients with rheumatoid arthritis, rheumatoid arthritis‐specific PPPY outpatient (5.1 vs. 3.9) and specialist visits (3.5 vs. 2.5), and disease‐specific ($4820 vs. $2592) costs were significantly higher in the bronchiectasis cohort (*p* < 0.05 for all comparisons).

**Conclusions:**

Bronchiectasis is associated with higher comorbid disease‐related healthcare resource utilization and costs and complicates the management of comorbid conditions.

## Graphical abstract

This study assessed the impact of bronchiectasis on managing patients with comorbid diseases, including COPD, asthma, and rheumatoid arthritis. The results suggest that bronchiectasis is associated with higher comorbid disease‐related healthcare resource utilization and direct medical costs, and complicates the management of comorbid conditions. Effective treatment of patients with bronchiectasis may reduce its impact on patients with comorbid disease.

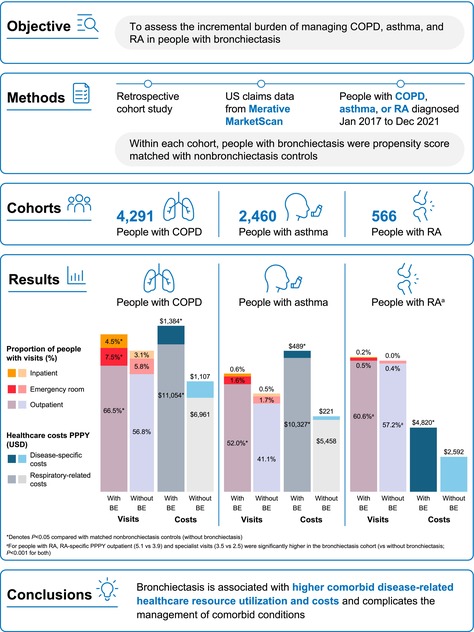

## 1 Introduction

Bronchiectasis is a chronic and progressive inflammatory lung disease characterized by permanent dilation of the bronchi and accompanied by cough, sputum production, and recurrent bronchial infection and exacerbations [1, 2]. Bronchiectasis imparts significant physical and psychosocial burden on patients, reducing their health‐related quality of life and imposing a substantial burden on the healthcare system [3, 4, 5].

Patients with bronchiectasis frequently present with related comorbidities, such as chronic obstructive pulmonary disease (COPD), asthma, and rheumatoid arthritis, which can further complicate disease management [1]. Previous US‐based real‐world studies estimated that 20%–46% of patients with bronchiectasis have comorbid COPD and 29%–32% have comorbid asthma [4, 6]. In the United States, a retrospective analysis found that rheumatoid arthritis was present in 4.6% of 14 798 patients with bronchiectasis [7].

Comorbidities further contribute to the disease burden, risk of mortality, and high healthcare resource utilization and costs in patients with bronchiectasis. An analysis of data from a national sample of South Korean patients with bronchiectasis (*N* = 14 823) found that the risk of all‐cause death was significantly increased in patients with bronchiectasis with comorbid COPD (HR = 1.24) or asthma (HR = 1.20) [8]. Another study of 1716 patients with bronchiectasis from clinical centers in Western Europe found that patients with comorbid rheumatoid arthritis or COPD had 2.1–3.1 times higher mortality rates compared with those with other comorbidities [9]. A retrospective analysis of claims data reported that annual levels of respiratory‐related healthcare utilization and expenditures in matched patients with bronchiectasis and comorbid COPD (*N* = 11 685) were 2.0–2.5 times higher than in patients with COPD only, and 2.4–3.5 times higher than in patients with bronchiectasis only [10].

Despite the known impact of comorbidities on disease burden and mortality in patients with bronchiectasis, the added impact of bronchiectasis on healthcare resource utilization and direct medical costs associated with the management of these comorbidities has not been evaluated in prior studies. The objective of this study was to assess the incremental burden of managing certain comorbid diseases, including COPD, asthma, and rheumatoid arthritis, in patients with bronchiectasis.

## 2 Material and Methods

### 2.1 Data Source

**FIGURE 1:**
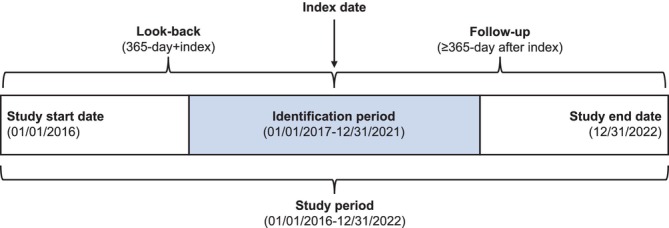
Study design.

This was a retrospective cohort study using administrative claims data from the Merative MarketScan Commercial Claims and Encounters Database with Medicare Supplement between January 1, 2016, and December 31, 2022 (Figure 1). The database contains deidentified medical and pharmaceutical claims data for 225 million unique patients from 300 employers and 40 health plans across the United States. Data included within this database are obtained from inpatient and outpatient medical claims, outpatient prescription claims, clinical utilization records, and healthcare expenditures. Data on both the employer‐paid portion and the Medicare‐paid portion of the payments are included in the database.

### 2.2 Study Population

Adult patients (≥ 18 years) diagnosed with COPD, asthma, or rheumatoid arthritis, as defined by *International Classification of Diseases, Tenth Revision, Clinical Modification* codes (see Table S1 for a full list of codes used), were included in the study if they met the following criteria: ≥ 2 outpatient claims with a diagnosis code (in any position) of interest dated ≥ 1 month apart, or ≥ 1 inpatient claim with a primary diagnosis of interest, between January 1, 2017, and December 31, 2021 (identification period). Within these cohorts, patients with a diagnosis of bronchiectasis were compared with matched nonbronchiectasis control patients using propensity score matching. A diagnosis of bronchiectasis was defined as: ≥ 2 outpatient claims for bronchiectasis dated ≥ 1 month apart or ≥ 1 inpatient claim with a primary diagnosis of bronchiectasis during the identification period; and no claim for cystic fibrosis in any setting. Matched nonbronchiectasis control patients were those patients within the COPD, asthma, or rheumatoid arthritis cohorts with no claim for bronchiectasis. The index date was a randomly selected date within the identification period after diagnosis criteria were met for each study group. All patients were required to have ≥ 12 months of continuous enrollment before index date (baseline period) and ≥ 12 months of potential follow‐up after index date.

### 2.3 Propensity Score Matching

Propensity scores were estimated to balance patient demographics and disease characteristics at baseline using a logistic regression model; variables used for matching were age at index, gender, index date, geographic location, payer type, Charlson comorbidity index, and disease‐specific hospitalizations at baseline. The nearest neighbor algorithm without replacement matching was implemented. Patients with bronchiectasis were matched with a 1:1 ratio with nonbronchiectasis control patients in the COPD and asthma cohorts; for the rheumatoid arthritis cohort, a 1:2 ratio was used to increase the precision in modeling estimates due to the small sample size of patients with bronchiectasis in this cohort. A standardized mean difference value below 0.2 was used to indicate that covariates were balanced between groups.

### 2.4 Outcomes

Demographic and clinical characteristics were assessed during the baseline period and included age, sex, index year, geographic region, type of health insurance, Charlson comorbidity index, and respiratory and nonrespiratory comorbidities. Proportions of patients and per person per year (PPPY) comorbid disease‐specific, respiratory‐related, and all‐cause inpatient, outpatient, emergency room (ER), general practitioner (GP), and specialist visits were reported. Disease‐specific visits were defined as any visit for which the primary diagnosis code was for the comorbidity of interest (i.e., COPD, asthma, or rheumatoid arthritis), while respiratory‐related visits were visits with a primary diagnosis code for respiratory disease or respiratory‐specific interventions/procedures, and all‐cause visits were defined as any visit related to any diagnosis. PPPY disease‐specific, respiratory‐related, and all‐cause direct medical costs were also reported. For patients with rheumatoid arthritis, disease‐specific and all‐cause visits and costs only were reported. Costs during the study period were inflated per the medical care component of the annual Consumer Price Index in 2022 USD.

### 2.5 Statistical Analyses

Categorical and continuous variables were assessed using descriptive statistics. Differences between groups were tested using Chi‐square test for categorical variables and Student's *t* test for continuous variables. Comparisons of healthcare resource utilization and costs between the bronchiectasis and matched nonbronchiectasis control groups were assessed with a Poisson model or a quasi‐Poisson regression model in the case of overdispersion for the COPD, asthma, and rheumatoid arthritis cohorts. For adjusted analyses, a stepwise approach was utilized to identify significant unbalanced baseline covariates (standardized mean difference ≥ 0.2) to be adjusted. No adjustments were made for multiplicity. *p* < 0.05 was considered significant.

## 3 Results

### 3.1 Study Population

Following propensity score matching, 4291 patients with COPD, 2460 with asthma, and 566 with rheumatoid arthritis, all of whom had bronchiectasis, and the corresponding nonbronchiectasis control patients, were included (Figure S1). In each cohort of patients, baseline demographics were balanced between patients with bronchiectasis and matched nonbronchiectasis controls (Table 1; Figure S2). The mean follow‐up ranged from 1.5 to 1.7 years across the cohorts. Certain comorbidities (e.g., respiratory infections, bronchitis, chronic rhinosinusitis, pneumonia, gastroesophageal reflux disease [GERD]) and symptoms (e.g., dyspnea and cough) were more common in patients with bronchiectasis vs. matched nonbronchiectasis controls. In the COPD cohort, the most common respiratory comorbidities or symptoms were dyspnea (59.0% vs. 45.7%), cough (47.2% vs. 28.2%), and acute lower respiratory symptoms (42.5% vs. 23.4%). In the asthma cohort, the most common respiratory comorbidities or symptoms were COPD (52.2% vs. 28.3%), dyspnea (51.4% vs. 35.1%), and cough (49.7% vs. 29.8%). GERD was a common nonrespiratory comorbidity in all cohorts (32.7% vs. 22.1% in COPD, 35.9% vs. 27.2% in asthma, and 33.6% vs. 23.9% in rheumatoid arthritis).

**TABLE 1:** Baseline demographics and clinical characteristics of matched patients with COPD, asthma, or rheumatoid arthritis, with or without bronchiectasis.

	Patients with COPD		Patients with asthma		Patients with rheumatoid arthritis	
	With bronchiectasis (*n* = 4291)	Without bronchiectasis (*n* = 4291)	With bronchiectasis (*n* = 2460)	Without bronchiectasis (*n* = 2460)	With bronchiectasis (*n* = 566)	Without bronchiectasis (*n* = 1132)
Length of follow‐up, mean (SD), years	1.5 (1.1)*	1.6 (1.1)	1.6 (1.0)*	1.7 (1.0)	1.5 (1.0)*	1.6 (1.1)
Age at index, mean (SD), years	76.2 (9.5)*	76.0 (9.8)	71.9 (12.1)	71.8 (12.3)	72.8 (10.3)	73.3 (10.4)
Female, *n* (%)	2537 (59.1)	2576 (60.0)	1726 (70.2)	1768 (71.9)	432 (76.3)	836 (73.9)
Index year, *n* (%)						
2021	2016 (47.0)	2134 (49.7)	1195 (48.6)	1232 (50.1)	288 (50.9)	558 (49.3)
2020	951 (22.2)	919 (21.4)	559 (22.7)	612 (24.9)	106 (18.7)	250 (22.1)
2019	646 (15.1)	619 (14.4)	351 (14.3)	331 (13.5)	74 (13.1)	158 (14.0)
2018	504 (11.7)	414 (9.6)	270 (11.0)	228 (9.3)	76 (13.4)	113 (10.0)
2017	174 (4.1)	205 (4.8)	85 (3.5)	57 (2.3)	22 (3.9)	53 (4.7)
US geographic region, *n* (%)						
North central	1972 (46.0)	2260 (52.7)	1029 (41.8)	1166 (47.4)	257 (45.4)	516 (45.6)
South	1292 (30.1)	1141 (26.6)	741 (30.1)	685 (27.8)	178 (31.4)	345 (30.5)
Northeast	769 (17.9)	660 (15.4)	489 (19.9)	398 (16.2)	89 (15.7)	178 (15.7)
West	255 (5.9)	227 (5.3)	197 (8.0)	208 (8.5)	42 (7.4)	93 (8.2)
Unknown	3 (0.1)	3 (0.1)	4 (0.2)	3 (0.1)	0	0
Medicare, *n* (%)	3837 (89.4)	3809 (88.8)	1901 (77.3)	1900 (77.2)	451 (79.7)	943 (83.3)
CCI, mean (SD)	3.7 (2.8)	3.7 (2.9)	3.0 (2.5)	3.0 (2.5)	4.0 (2.5)*	3.9 (2.8)
Select comorbidities and symptoms, *n* (%)						
COPD	4291 (100)	4291 (100)	1285 (52.2)*	696 (28.3)	260 (45.9)*	207 (18.3)
Dyspnea	2531 (59.0)*	1963 (45.7)	1265 (51.4)*	864 (35.1)	290 (51.2)*	295 (26.1)
Cough	2025 (47.2)*	1208 (28.2)	1223 (49.7)*	732 (29.8)	218 (38.5)*	212 (18.7)
Other diseases of the respiratory system^a^	2025 (47.2)*	1144 (26.7)	932 (37.9)*	397 (16.1)	229 (40.5)*	164 (14.5)
Acute lower respiratory infections	1823 (42.5)*	1004 (23.4)	879 (35.7)*	464 (18.9)	186 (32.9)*	156 (13.8)
Bronchitis	1499 (34.9)*	817 (19.0)	707 (28.7)*	399 (16.2)	128 (22.6)*	104 (9.2)
Emphysema	1407 (32.8)*	1009 (23.5)	318 (12.9)*	140 (5.7)	70 (12.4)*	67 (5.9)
GERD	1404 (32.7)*	948 (22.1)	884 (35.9)*	670 (27.2)	190 (33.6)*	271 (23.9)
Pneumonia	1378 (32.1)*	678 (15.8)	630 (25.6)*	228 (9.3)	142 (25.1)*	102 (9.0)
Asthma	1255 (29.2)*	765 (17.8)	2460 (100)	2460 (100)	161 (28.4)*	147 (13.0)
Acute upper respiratory infections	765 (17.8)*	622 (14.5)	547 (22.2)	540 (22.0)	86 (15.2)	178 (15.7)
Chronic rhinosinusitis	558 (13.0)*	278 (6.5)	523 (21.3)*	296 (12.0)	74 (13.1)*	75 (6.6)
Tobacco dependence	462 (10.8)*	829 (19.3)	99 (4.0)	103 (4.2)	31 (5.5)	54 (4.8)
NTMLD	333 (7.8)*	10 (0.2)	182 (7.4)*	7 (0.3)	38 (6.7)*	3 (0.3)
Rheumatoid arthritis	276 (6.4)*	152 (3.5)	177 (7.2)*	103 (4.2)	566 (100)	1132 (100)
Sarcoidosis	94 (2.2)*	34 (0.8)	79 (3.2)*	24 (1.0)	15 (2.7)*	10 (0.9)
CVID	86 (2.0)*	5 (0.1)	75 (3.0)*	17 (0.7)	18 (3.2)*	2 (0.2)
Sjögren syndrome	60 (1.4)*	27 (0.6)	55 (2.2)*	19 (0.8)	40 (7.1)*	46 (4.1)
Systemic sclerosis	19 (0.4)*	3 (0.1)	9 (0.4)	5 (0.2)	14 (2.5)*	9 (0.8)

Abbreviations: CCI, Charlson comorbidity score; COPD, chronic obstructive pulmonary disease; CVID, common variable immunodeficiency; GERD, gastroesophageal reflux disease; *ICD‐10‐CM*, *International Classification of Diseases, Tenth Revision, Clinical Modification* codes; NTMLD, nontuberculous mycobacterial lung disease.

* Denotes standardized mean difference > 0.02 or *p* < 0.05 compared with matched nonbronchiectasis control patients.

^a^ Distinct claims category captured by *ICD‐10‐CM* codes: J96, J98, J99.

### 3.2 Comorbid Disease‐Specific Healthcare Resource Utilization

**FIGURE 2:**
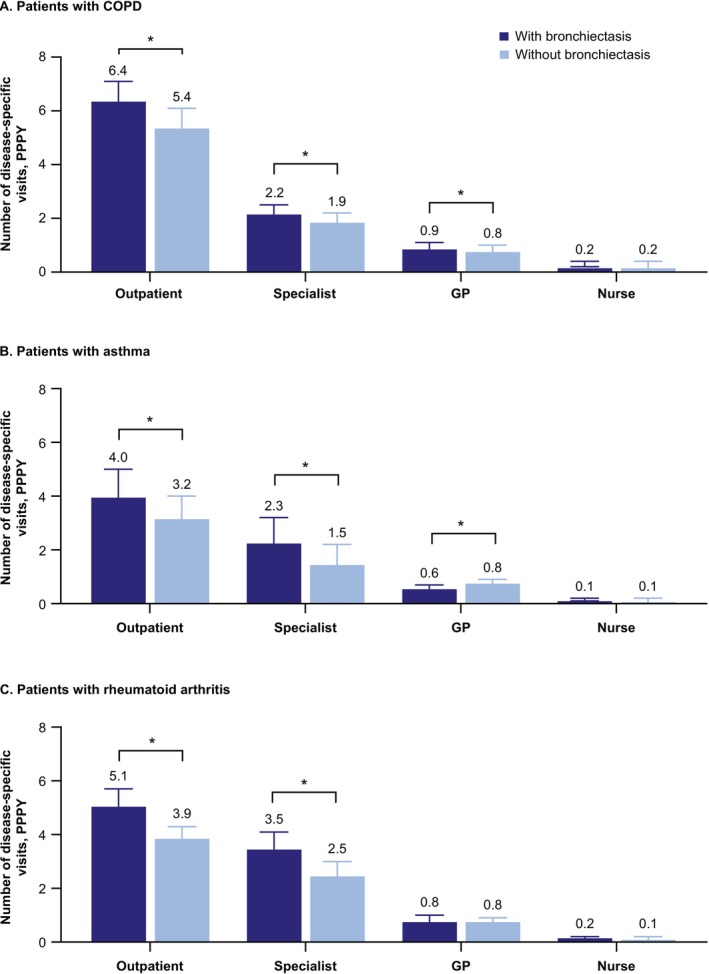
Mean (95% CI) number of disease‐specific visits^a^ PPPY in patients with (A) COPD, (B) asthma, and (C) rheumatoid arthritis with at least one disease‐specific visit. *Denotes *p* < 0.05 compared with matched nonbronchiectasis control patients. ^a^Outpatient includes GP, specialist, and nurse visits, as well as additional services not shown. CI, confidence interval; COPD, chronic obstructive pulmonary disease; ER, emergency room; GP, general practitioner; PPPY, per patient per year.

For patients with COPD, proportions with COPD‐related outpatient visits (66.5% vs. 56.8%, *p* < 0.001), ER visits (7.5% vs. 5.8%, *p =* 0.001), and inpatient visits (4.5% vs. 3.1%, *p =* 0.001) were 1.2–1.5 times higher within the bronchiectasis cohort than nonbronchiectasis control cohort (Table 2). COPD‐related PPPY outpatient visits were more frequent (6.4 vs. 5.4, *p* < 0.001) in patients with bronchiectasis. This category included specialist and GP visits, which individually were also more frequent in patients with bronchiectasis (*p* < 0.01 for both) (Figure 2).

**TABLE 2:** Healthcare resources utilization among patients with COPD, asthma, and rheumatoid arthritis, with or without bronchiectasis, with at least one disease‐specific or respiratory‐related^a^ healthcare visit during follow‐up.

	Patients with COPD		Patients with asthma		Patients with rheumatoid arthritis	
	With bronchiectasis (*n* = 4291)	Without bronchiectasis (*n* = 4291)	With bronchiectasis (*n* = 2460)	Without bronchiectasis (*n* = 2460)	With bronchiectasis (*n* = 566)	Without bronchiectasis (*n* = 1132)
With a disease‐specific healthcare visit, *n* (%)						
Outpatient	2854 (66.5)*	2436 (56.8)	1279 (52.0)*	1011 (41.1)	343 (60.6)	648 (57.2)
Inpatient	191 (4.5)*	134 (3.1)	14 (0.6)	12 (0.5)	1 (0.2)	0
ER	323 (7.5)*	249 (5.8)	40 (1.6)	41 (1.7)	3 (0.5)	5 (0.4)
With respiratory‐related healthcare visits, *n* (%)						
Outpatient	3861 (90.0)*	3279 (76.4)	2212 (89.9)*	1818 (73.9)	N/A	N/A
Inpatient	930 (21.7)*	605 (14.1)	390 (15.9)*	180 (7.3)	N/A	N/A
ER	761 (17.7)*	575 (13.4)	360 (14.6)*	214 (8.7)	N/A	N/A

Abbreviations: COPD, chronic obstructive pulmonary disease; ER, emergency room.

* Denotes *p* < 0.05 compared with matched nonbronchiectasis control patients.

^a^ Respiratory‐related healthcare visits were not assessed in patients with rheumatoid arthritis as it is not a respiratory condition.

For patients with asthma, proportions with asthma‐related outpatient visits were 1.3 times higher (52.0% vs. 41.1%; *p* < 0.001) within the bronchiectasis cohort than the nonbronchiectasis control cohort. Asthma‐related PPPY outpatient visits were 1.3 times higher (4.0 vs. 3.2, *p* < 0.001) in patients with bronchiectasis, with specialist and GP visits similarly more frequent (*p* < 0.01 for both).

For patients with rheumatoid arthritis, rheumatoid arthritis‐specific PPPY outpatient visits were 1.3 times higher (5.1 vs. 3.9) within the bronchiectasis cohort than the nonbronchiectasis control cohort, with specialist visits (3.5 vs. 2.5) accounting for the majority of visits (*p* < 0.001 for both).

### 3.3 Respiratory‐Related Healthcare Resource Utilization

**FIGURE 3:**
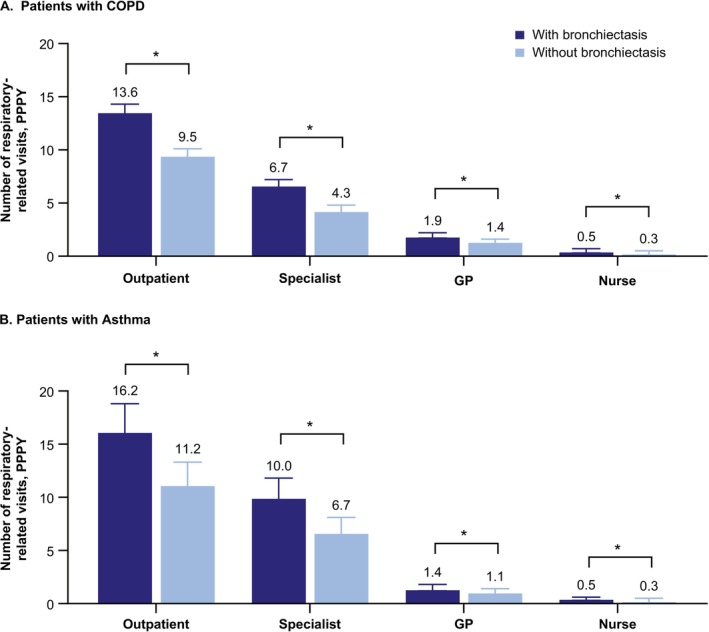
Mean (95% CI) number of respiratory‐related visits^a^ PPPY in patients with (A) COPD and (B) asthma with at least one respiratory‐related visit. *Denotes *p* < 0.05 compared with matched nonbronchiectasis control patients. ^a^Outpatient includes GP, specialist, and nurse visits, as well as additional services not shown. CI, confidence interval; COPD, chronic obstructive pulmonary disease; ER, emergency room; GP, general practitioner; PPPY, per patient per year.

For patients with COPD, proportions with respiratory‐related outpatient visits (90.0% vs. 76.4%), inpatient visits (21.7% vs. 14.1%), and ER visits (17.7% vs. 13.4%) were 1.2–1.5 times higher within the bronchiectasis cohort than the nonbronchiectasis control cohort (*p* < 0.001 for all) (Table 2). Respiratory‐related PPPY outpatient visits were 1.4 times higher (13.6 vs. 9.5, *p* < 0.001) in patients with bronchiectasis. This category included specialist, GP, and nurse visits, which individually were also more frequent in patients with bronchiectasis (*p* < 0.001 for all) (Figure 3).

For patients with asthma, proportions with respiratory‐related outpatient visits (89.9% vs. 73.9%), inpatient visits (15.9% vs. 7.3%), and ER visits (14.6% vs. 8.7%) were 1.2–2.2 times higher within the bronchiectasis cohort than the nonbronchiectasis control cohort (*p* < 0.001 for all). Respiratory‐related PPPY outpatient visits were 1.4 times higher (16.2 vs. 11.2) in patients with bronchiectasis, with specialist visits (10.0 vs. 6.7) accounting for the majority of visits (*p* < 0.001 for both).

### 3.4 All‐Cause Healthcare Resource Utilization

For patients with COPD, proportions with all‐cause outpatient visits (97.6% vs. 96.1%, *p* < 0.001), ER visits (45.7% vs. 42.6%, *p* = 0.003), and inpatient visits (40.9% vs. 36.7%, *p* < 0.001) were higher within the bronchiectasis cohort than the nonbronchiectasis control cohort. All‐cause PPPY outpatient visits were higher (41.4 vs. 36.9) in patients with bronchiectasis, including specialist (27.3 vs. 24.7) and GP visits (7.8 vs. 7.2) (*p* < 0.001 for all).

For patients with asthma, proportions with all‐cause inpatient visits were 1.3 times higher (32.1% vs. 25.0%, *p* < 0.001) within the bronchiectasis cohort than the nonbronchiectasis control cohort. All‐cause PPPY outpatient visits were more frequent (50.4 vs. 46.0) in patients with bronchiectasis, with specialist visits accounting for the majority of these (34.6 vs. 31.4) (*p* < 0.001 for both).

For patients with rheumatoid arthritis, proportions with all‐cause inpatient (39.6% vs. 29.7%, *p* < 0.001) and ER visits (44.0% vs. 37.5%, *p* = 0.009) were higher within the bronchiectasis cohort than the nonbronchiectasis control cohort. All‐cause PPPY outpatient visits were more frequent (53.0 vs. 48.5, *p* = 0.026) in patients with bronchiectasis, with specialist visits accounting for the majority of these (38.6 vs. 34.9, *p* = 0.044).

### 3.5 Direct PPPY Medical Costs

**FIGURE 4:**
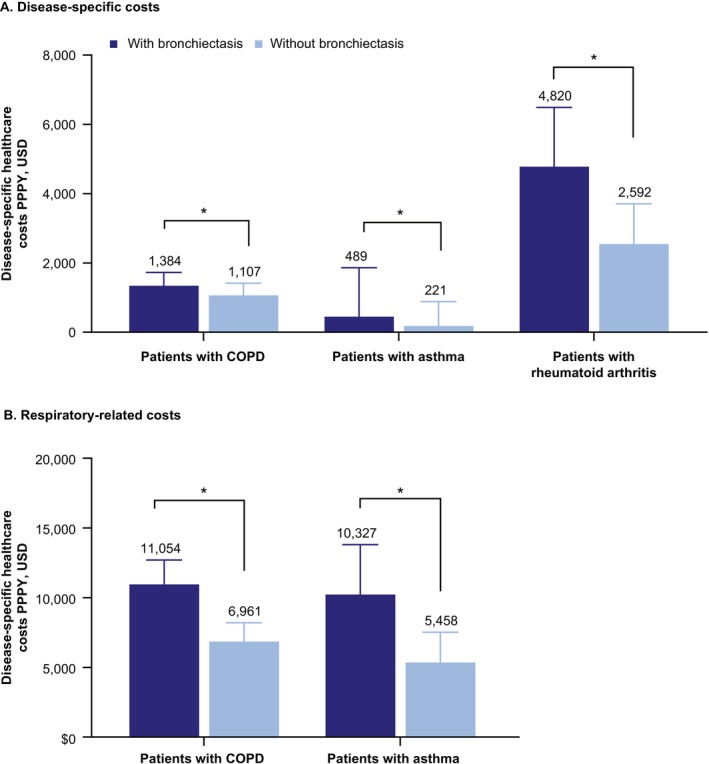
Mean (95% CI) (A) disease‐specific and (B) respiratory‐related healthcare costs^a^ PPPY in patients with COPD, asthma, and rheumatoid arthritis with at least one disease‐specific visit. *Denotes *p* < 0.05 compared with matched nonbronchiectasis control patients. ^a^Costs are total disease‐specific medical costs and do not include pharmacy costs. Costs during the study period were inflated per the medical care component of the annual Consumer Price Index in 2022 USD. CI, confidence interval; COPD, chronic obstructive pulmonary disease; PPPY, per patient per year; RA, rheumatoid arthritis.

For patients with COPD, COPD‐specific costs were 1.3 times higher ($1384 vs. $1107, *p* = 0.002), and respiratory‐related costs 1.6 times higher ($11 054 vs. $6961, *p* < 0.001), within the bronchiectasis cohort than the nonbronchiectasis control cohort (Figure 4). All‐cause medical costs ($39 686 vs. $34 184) and pharmacy costs ($7872 vs. $5732) were also higher in the bronchiectasis cohort (*p* < 0.001 for both).

In patients with asthma, asthma‐specific costs were 2.2 times higher ($489 vs. $221, *p* < 0.001), and respiratory‐related medical costs 1.9 times higher ($10 327 vs. $5458, *p* < 0.001), within the bronchiectasis cohort than the nonbronchiectasis control cohort. All‐cause medical costs ($50 265 vs. $43 497, *p* = 0.007) and pharmacy costs ($11 922 vs. $8995, *p* < 0.001) were also higher in the bronchiectasis cohort.

For patients with rheumatoid arthritis, rheumatoid arthritis‐specific costs were 1.9 times higher within the bronchiectasis cohort ($4820 vs. $2592, *p* < 0.001). All‐cause ($73 058 vs. $63 633, *p* = 0.096) and rheumatoid arthritis‐specific outpatient ($6787 vs. $3821, *p* = 0.002) costs were also higher within the bronchiectasis cohort.

## 4 Discussion

This study expands on prior studies characterizing the burden of bronchiectasis [4, 5, 11] and is the first to show the incremental burden of managing certain comorbid diseases in patients with bronchiectasis. For patients with COPD, asthma, or rheumatoid arthritis, the presence of bronchiectasis significantly increased comorbid disease‐specific healthcare resource utilization and medical costs across different healthcare services. An earlier retrospective analysis of US claims data from 11 685 matched patients with bronchiectasis, COPD, or comorbid bronchiectasis and COPD reported that healthcare resource utilization and costs in those patients with comorbid bronchiectasis and COPD were greater than the sum of the corresponding values for either condition alone [10]. Our study expands on this analysis by including additional comorbidities, such as asthma and rheumatoid arthritis, and evaluating the disease‐specific costs associated with these comorbidities. Notably for patients with COPD or asthma, disease‐specific costs were not as large, and the difference between patients with and without bronchiectasis not as great as those for rheumatoid arthritis. However, respiratory‐related costs were higher than disease‐specific costs overall and were especially high in patients with bronchiectasis (ranging from 1.6 to 1.9 times greater than in patients without bronchiectasis). The coexistence of multiple respiratory comorbidities and the overlap between their symptoms (i.e., cough, dyspnea, and exacerbations) make respiratory‐related costs a more accurate reflection of overall disease burden than costs linked to disease‐specific codes alone. A strength of the methodology was the propensity score matching technique used to balance the patient demographics within the study cohorts. After adjusting for patient demographics, patients with bronchiectasis had a higher prevalence of comorbidities at baseline than those without bronchiectasis across all cohorts, with dyspnea, cough, and GERD being common, which is consistent with previous analyses [11].

Previous studies have shown that patients with comorbid bronchiectasis and asthma have higher rates of pulmonary exacerbations than patients with either condition only [12, 13]. A study in South Korea of 50 matched patients with asthma and comorbid bronchiectasis or asthma alone reported that the annual incidence of asthma exacerbations and associated steroid use and ER visits were higher in patients with comorbid bronchiectasis than without [13]. Similarly, a study in Italy of 106 patients with bronchiectasis reported an increased risk of bronchiectasis exacerbations in those patients who had comorbid asthma [12]. In patients with bronchiectasis, a higher frequency of pulmonary exacerbations has been associated with greater healthcare resource utilization and costs [4].

There may be an association or causal relationship between rheumatoid arthritis and bronchiectasis, with both associated with underlying chronic inflammation [14]. Although patients with rheumatoid arthritis were shown to have approximately twice the risk of developing bronchiectasis as matched nonbronchiectasis controls in one study, very little is known about the added burden of bronchiectasis in rheumatoid arthritis [15]. A meta‐analysis of studies published between 2003 and 2015 demonstrated the high medical costs of treating rheumatoid arthritis in the United States, ranging from $12 509 to $36 053 annually [16]. This study adds to the previous analyses by suggesting that bronchiectasis nearly doubled the disease‐specific costs associated with managing rheumatoid arthritis.

The results of this study underscore the need for effective diagnosis and management of bronchiectasis to reduce the overall disease burden of COPD, asthma, and rheumatoid arthritis in patients and on the healthcare system.

This study had several limitations that should be considered. As with all claims‐based studies, there exists a misclassification bias for the selection of the eligible population due to nonaccurate diagnosis codes or misspecification of diagnosis codes in the claims data; this misclassification bias extends to the attribution of healthcare resource utilization and costs. To account for these differences, disease‐specific, respiratory‐related and all‐cause healthcare resource utilization and costs were assessed, with patterns generally consistent across categories. As patients with COPD or asthma who have comorbid bronchiectasis often have overlapping respiratory symptoms, disease‐specific costs may be an underestimate due to the likelihood of miscoded or undercoded claims. Although propensity score matching was performed to balance patient characteristics, baseline comorbidities between the matched patient groups were imbalanced, which may have led to residual confounding. Additionally, unmeasured variables that could not be accounted for in the propensity score matching may have also influenced the residual confounding. Some information that may be relevant for understanding disease severity and other characteristics of patients, such as spirometry results, race/ethnicity, smoking status or history, alcohol consumption, socioeconomic status, body mass index, health‐related quality of life, literacy, and access to care, are not available using this claims data source. Patients in the study sample were predominantly older adults (77%–89% of patients had Medicare Supplemental Insurance and mean age was approximately 72–76 years), which may limit the generalizability of the asthma‐related results due to its younger demographic profile. The MarketScan Medicare Supplemental database captures only those services that generated a claim through the employer‐sponsored supplemental plan and does not include full Medicare claims for all covered services under Parts A and B. Additionally, the study is limited to patients enrolled in commercial insurance plans which may not be representative of patients with Medicare coverage only. Although a higher proportion of patients had an index date in 2021, this likely reflects structural characteristics of the data source and study design rather than an increase in diagnoses.

## 5 Conclusions

Bronchiectasis complicates the management of patients with comorbid disease and is associated with higher comorbid disease‐related healthcare resource utilization and costs. Effective treatment of patients with bronchiectasis could help to reduce its impact on patients with comorbid disease.

## Author Contributions

Conceptualization: M.M., F.T., J.F., J.F., M.L., S.F., M.H., R.T.S.; Methodology: M.M., F.T., J.F., J.F., M.L., S.F., M.H., R.S.; Validation: F.T., M.H.; Formal analysis, F.T., M.H.; Writing – original draft: M.M., F.T., J.F., J.F., M.L., S.F., M.H., R.S.; Writing – review and editing: M.M., F.T., J.F., J.F., M.L., S.F., M.H., R.S.

## Funding

This study was funded by Insmed Incorporated.

## Ethics Statement

Ethics approval was not required because this was a retrospective database study using deidentified data.

## Conflicts of Interest

Fei Tang and Mahroz Haider were employees of Cytel, a paid consultant for Insmed Incorporated, at the time of the study. John Fastenau, Joseph Feliciano, Maitreyee Mohanty, Melanie Lauterio, and Sebastian Fucile are employees and shareholders of Insmed Incorporated. Ruxana Sadikot has received grants from VA Merit Review and RO1 from NIH NHLBI, and honoraria from the American Physician Institute.

## Supporting information


**Figure S1:** Patient attrition for the (A) COPD, (B) asthma, and (C) rheumatoid arthritis cohorts.
**Figure S2:** Distribution of propensity scores before (left panel) and after matching (right panel) for the (A) COPD, (B) asthma, and (C) rheumatoid arthritis cohorts.
**Table S1:** Summary of *International Classification of Diseases, Tenth Revision, Clinical Modification* codes.

## Data Availability

Research data are not shared.
