# Supplementary material for: Impact of Bronchiectasis on Healthcare Resource Utilization and Direct Medical Costs of Managing Comorbid Chronic Obstructive Pulmonary Disease, Asthma, and Rheumatoid Arthritis in the United States
Source: Clin Respir J. 2025 Dec 25;19(12):e70150. doi: 10.1111/crj.70150 (PMC12740571; doi:10.1111/crj.70150)
Supplement: Supplementary file 1 — Figure S1: Patient attrition for the (A) COPD, (B) asthma, and (C) rheumatoid arthritis cohorts. Figure S2: Distribution of propensity scores before (left panel) and after matching (right panel) for the (A) COPD, (B) asthma, and (C) rheumatoid arthritis cohorts. Table S1: Summary of International Classification of Diseases, Tenth Revision, Clinical Modification codes. [file CRJ-19-e70150-s001.docx]

**Supplementary Figure 1. Patient attrition for the (A) COPD, (B) asthma, and (C) rheumatoid arthritis cohorts**


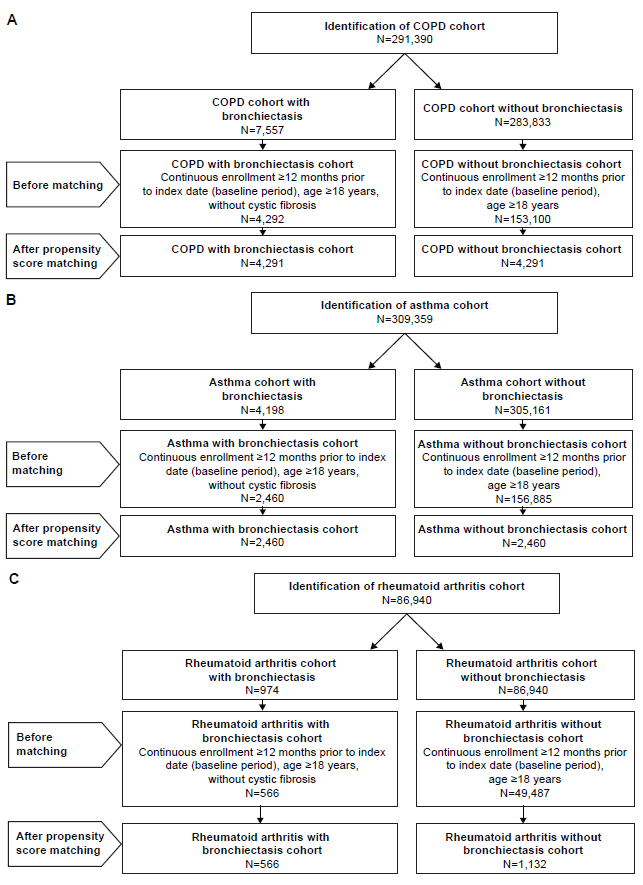


COPD, chronic obstructive pulmonary disease

**Supplementary Figure 2. Distribution of propensity scores before (left panel) and after matching (right panel) for the (A) COPD, (B) asthma, and (C) rheumatoid arthritis cohorts**


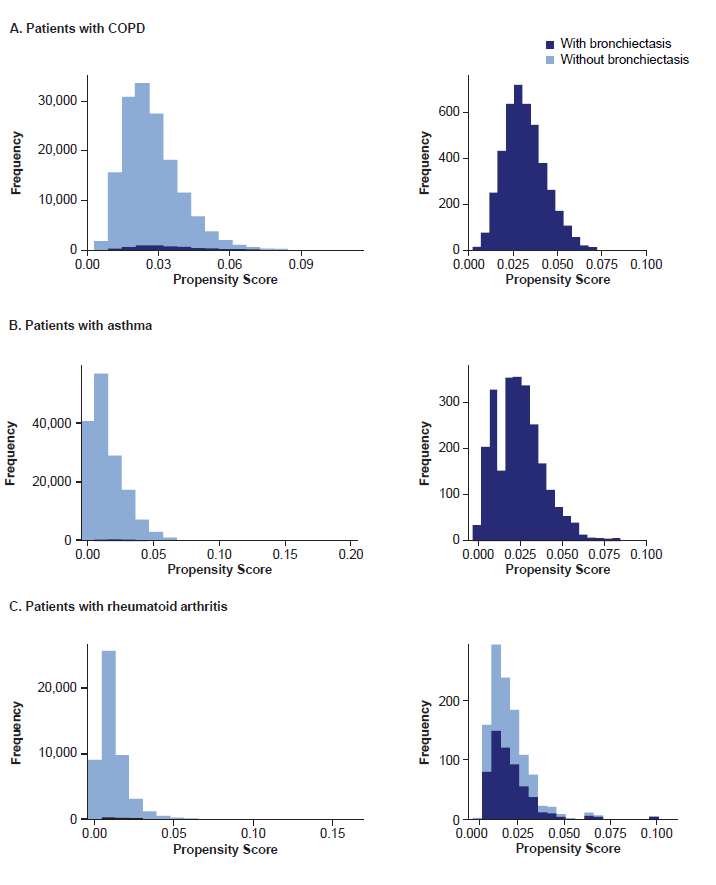


Distribution of propensity scores for the study cohorts before and after matching. The before matching distribution illustrates baseline differences between the bronchiectasis and nonbronchiectasis cohorts. After matching, the figures illustrate the overlapping distribution of propensity scores

COPD, chronic obstructive pulmonary disease

**Supplementary Table 1. Summary of *International Classification of Diseases, Tenth Revision, Clinical Modification* codes**

| **Condition** | **Description** | ***ICD-10-CM*** |
| --- | --- | --- |
| Asthma | Asthma | J45 |
| Bronchiectasis | Bronchiectasis with acute lower respiratory infection | J47.0 |
|  | Bronchiectasis with (acute) exacerbation | J47.1 |
|  | Bronchiectasis, uncomplicated | J47.9 |
| COPD | Simple chronic bronchitis | J410 |
|  | Mucopurulent chronic bronchitis | J411 |
|  | Mixed simple and mucopurulent chronic bronchitis | J418 |
|  | Unspecified chronic bronchitis | J42 |
|  | Unilateral pulmonary emphysema [MacLeod's syndrome] | J430 |
|  | Panlobular emphysema | J431 |
|  | Centrilobular emphysema | J432 |
|  | Other emphysema | J438 |
|  | Emphysema, unspecified | J439 |
|  | Chronic obstructive pulmonary disease with (acute) lower respiratory infection | J440 |
|  | Chronic obstructive pulmonary disease with (acute) exacerbation | J441 |
|  | Chronic obstructive pulmonary disease, unspecified | J449 |
| Cystic fibrosis | Cystic fibrosis with pulmonary manifestations | E84.0 |
|  | Cystic fibrosis with other manifestations | E84.8 |
|  | Meconium ileus in cystic fibrosis | E84.11 |
|  | Cystic fibrosis with other intestinal manifestations | E84.19 |
|  | Cystic fibrosis, unspecified | E84.9 |
| Rheumatoid arthritis | Rheumatoid arthritis | M05, M06 |

COPD, chronic obstructive pulmonary disease; *ICD-10-CM, International Classification of Diseases, Tenth Revision, Clinical Modification*
